# Analysis of the bacterial community in chronic obstructive pulmonary disease sputum samples by denaturing gradient gel electrophoresis and real-time PCR

**DOI:** 10.1186/1471-2466-14-179

**Published:** 2014-11-18

**Authors:** Dachang Wu, Chenxia Hou, Yanxia Li, Zinan Zhao, Jianjun Liu, Xin Lu, Xueqi Shang, Yi Xin

**Affiliations:** Biotechnology Department, Dalian Medical University, 9 Western Section, Lvshun South Street, Dalian, P.R. China; Respiratory Medicine Department, The First affiliated hospital of Dalian Medical University, 222 ZhongShan Road, Dalian, P.R. China

## Abstract

**Background:**

The Global Initiative defines COPD for chronic obstructive lung disease as an entirely preventable and treatable disease characterized by sputum production, bacterial colonisation, neutrophilic bronchial airway inflammation and poor health status. The World Health Organization (WHO) estimates that COPD will become the fourth-most common cause of death worldwide, just behind ischemic heart disease, cerebrovascular disease and HIV/AIDS, by 2030. The aim of this study was to determine the main structure feature of sputum potentially pathogenic microorganisms in subjects with COPD during the clinical stable state.

**Methods:**

We employed a molecular genetics-based investigation of the bacteria community, including DNA isolation, PCR amplification and DGGE profiling. PCR-denaturing gradient gel electrophoresis (DGGE) with universal primers targeting the V3 region of the 16S rRNA gene was employed to characterize the overall COPD patient sputum microbiota composition, and some excised gel bands were cloned for sequencing. Real-time PCR was further utilized to quantitatively analyze the subpopulation of microbiota using group-specific primers targeting *Streptococcus pneumoniae*, *Klebsiella pneumoniae*, *Pseudomonas aeruginosa*.

**Results:**

The DGGE profiles of two groups displayed significant differences between COPD and healthy groups (*P* < 0.05). Real-time PCR revealed significant increases of *Streptococcus pneumoniae*, *Klebsiella pneumoniae* and *Pseudomonas aeruginosa* (*P* < 0.05) in the COPD group compared with the healthy group.

**Conclusion:**

This study revealed strong relationship between alterations of sputum microbiota and COPD. By determining the content of several types of bacteria, we can provide evidence to aid in the diagnosis and treatment of COPD.

## Background

Chronic obstructive pulmonary disease (COPD) is categorized by the severity of the airflow obstruction based on the patient’s predicted forced expiratory volume in one second [1, 2]. COPD involves a variety of pathological processes, such as chronic bronchitis, chronic bronchiolitis and emphysema [3]. These pathological processes may occur individually or in combination. Acute exacerbations are common and occur once to three times per year, which leads to most of the observed morbidity and mortality among patients [3, 4]. Bronchial infection is the most common cause and patients with frequent exacerbations appear to have a more rapid decline in quality of life [5]. In addition, acute exacerbations account for frequent medical visits, hospital admission [6] and death in patients with COPD. A longer-term legacy of an acute exacerbation that is also seen is the persistence of bacteria in the lower airway after an acute exacerbation, which is associated with increased inflammation and can lead to the progressive loss of lung function.

Most COPD exacerbations are infectious and largely either bacterial, though viral pathogens have also been isolated during exacerbations [7]. *Haemophilus influenza. Strep. pneumoniae*, *Moraxella catarrhalis* and *Pseudomonas aeruginosa*[8, 9] are the most frequently isolated bacterial agents during exacerbations. A large number of studies have indicated that one or more pathogens can be isolated from patients’ sputum during exacerbations. In a longitudinal study performed by Sethi and his colleagues of sputum microbiology in moderate to severe category COPD patients with an average FEV1 of 47, COPD exacerbations were commonly observed with new bacterial strains: *Haemophilus influenza* or *Moraxella catarrhalis*. This study also demonstrated that a great many instances where sputum microbiology were culture positive even without a clear diagnosis of exacerbation [10].

David Soll [11] defined polymicrobial diseases as those diseases that can occur with organisms from different kingdoms, from different genera within a kingdom and finally from different substrains within a strain. The classification of polymicrobial infection refers to polyviral infections, polybacterial infections, viral and bacterial infections, polymicrobial mycotic infections, and infections resulting from microbe-induced immunosuppression. Typically, polymicrobial infection can lead to a more severe situation than those originated from a single etiologic agent. Polymicrobial infections commonly occur in the oral cavity, the upper and lower airway, and the gastrointestinal tract. More and more diseases have already been defined as polymicrobial. However, there are still many diseases that remain to be proven as polymicrobial. In some cases, polymicrobial diseases are not sensitive to antibiotic treatment. Thus, it is important to identify the etiologic agents for polymicrobial infection and the part that each agent may play in the course of disease.

This study was aimed at characterizing the complex microbial diversity profile of the patient with COPD and healthy individuals. PCR-DGGE analysis provided insight into the overall microbiota community, whereas real-time PCR was used to quantify *Streptococcus pneumoniae*, *Klebsiella pneumoniae*, and *Pseudomonas aeruginosa* to observe changes at the specific genus level.

## Methods

### Ethics considerations

The study was approved by the Medical Ethics Committee of the First Affiliated Hospital of Dalian Medical University (Permit Number: KY2012–36).

### Sputum specimen collection

Twenty subjects (10 COPD patients and 10 healthy volunteers) aged 60–80 years participated in the study. The clinical samples were diagnosed and obtained from the First Affiliated Hospital of Dalian Medical University from August to October 2012. All patients had not received any antibiotic treatment. Written informed consent was obtained from all participants who were treated in compliance with the Helsinki Declaration on the participation of human subjects in medical research. Prior to the investigation, sputum samples were stored at – 80°C.

### DNA extraction

Prior to DNA extraction, all of the sputum samples were digested and decontaminated with N-acetyl-L-cysteine(NALC)-NaOH. Two volumes of NALC-NaOH solution (4% NaOH, 1.45% Na-citrate, and 0.5% NALC) were mixed with each sputum specimen in a sterilized test tube for digestion. The mixture was cultured at room temperature for 15 minutes with gentle shaking. Ten volumes of 6.7 mM phosphate buffer solution (PBS, pH 7.4) were added and the mixture centrifuged at 3,000 x g for 15 minutes at room temperature. The supernatant was discarded, and the pellet washed twice with PBS. Total bacterial DNA was extracted using QIAmp DNA Mini and Blood Mini kits (Qiagen, CA, USA) according to the manufacturer’s instructions. Briefly, a 100-μl aliquot of the decontaminated sputum specimen was mixed with an equal volume of deionized water and centrifuged for 10 min at 14,000 × g. The pellet was resuspended in ATL buffer (Qiagen, CA, USA) containing 1 mg/ml proteinase K and incubated at 56°C for 60 min. Subsequently, two cycles of freeze-thawing were performed to lyse the mycobacterial cells. DNA was purified and collected for further detection. The integrity of the nucleic acids was determined visually by 1% agarose gels electrophoresis containing ethidium bromide. DNA extraction and PCR amplification were performed in a specific PCR diagnosis room to prevent cross-contamination of nucleic acids.

### PCR amplification

Primers targeting the variable V3 region of 16S rRNA gene were applied, and the procedure performed following our previous publicized method [12]. Each 50 μl of the PCR reaction mixture contained 20 pmol of each primer, 20 mM of dNTP mixture, 5 μl of 10 × Ex Taq buffer (Mg^2+^ plus), 5 μl of 1% BSA, 2.5 U of Ex Taq DNA polymerase (TakaRa, Japan), and 2 μl of DNA template (approximately 200 ng). PCR amplification was performed in an automated thermocycler (Thermo USA). The PCR program was as follows: 94°C for 5 min; 30 cycles of 94°C for 30 s, 54°C for 30 s, and 72°C for 30 s; and lastly, 72°C for 7 min. The size of the obtained amplicons was checked through electrophoresis in a 2% agarose gel containing ethidium bromide. The presence of a 200-bp band on the agarose gel indicated successful amplification.

### Denaturing gradient gel electrophoresis and DGGE profiles analysis

PCR-based DGGE analysis was conducted to rapidly detect microbial community structure, followed by subsequent confirmation by qPCR and DNA sequencing. Briefly, DGGE analysis was performed by a Universal Mutation Detection System (Bio-Rad, USA) with an 8% polyacrylamide gel containing a 35–65% gradient of urea and formamide (a 100% denaturing solution contained 40% [v/v] formamide and 7.0 mM urea) as reported [13]. The ratio of acrylamide to bisacrylamide was 37.5:1. The electrophoresis was run at 200 V for 10 min, followed by a constant temperature of 60°C at 65 V for 7 hours. The gels were stained with ethidium bromide solution for 60 min, washed with deionized water, and viewed with a Gel Documentation System (Bio-Rad, USA) and photographed on a UV transilluminator.

The DGGE gel images were analyzed using Phoretix 1D (Single Gel Dendrogram) software (Phoretix, Newcastle upon Tyne, UK) [14]. The analysis took into account the number of bands, their gray intensity and the similarity of DGGE profiles. Similarities were displayed graphically as a dendrogram. The clustering algorithm that was used to calculate the dendrogram was an unweighted pair group method with arithmetic averages (UPGMA) [15]. The Shannon–Weaver index of diversity (H’) has been used to determine the diversity of taxa present in gut microbiota from COPD and healthy groups [16]. As the data were nonuniformly distributed, a nonparametric statistical analysis was performed with the Mann–Whitney *U* test, where a probability value *P* < 0.05 was considered as statistically significant. The nonparametric statistical analysis was performed using SPSS (version 11.5). The evenness (E), which reflected uniformity of the bacterial species distribution, was also computed. The H’ and E value were calculated with the following respective formulas: Shannon–Weaver index (H’) = -∑(*Pi*) (In *Pi*); evenness (E) = H’/In S. *Pi* is the proportion of species/bands for the ith species/band in the sample [17]. S is the number of bands.

### DNA sequencing

To identify some separated and specific bands, a sterile scalpel was used to cut out the bands from polyacrylamide gel under UV illumination. The gel fragments were washed once in 200 μl of sterile deionized water and kept in 50 μl of sterile water overnight at 4°C for diffusion. The extracted gel mix was heated at 90°C for 10 min, and 4 μl of the solution was taken as the DNA template for re-amplifying by PCR using the original primers without a GC clamp. The PCR program was the same as described previously. After purification, the PCR products were cloned into the PMD18-T Easy vector (TaKaRa, Japan), transformed into competent *Escherichia coli* Nova blue cells, and screened for positive plasmid insertions according to the manufacturer’s instructions. A second PCR was utilized to confirm the successful construction of the recon. The obtained PCR products were purified and sent for sequencing (TaKaRa, Japan). The sequence data were compared directly with those in GenBank by BLAST search (NCBI).

### Real-Time PCR

*Streptococcus pneumoniae*, *Klebsiella pneumoniae*, *Pseudomonas aeruginosa*, the important member groups with their unique characteristics in the sputum, were quantified by real-time PCR using group-specific primers. The reactions were performed in real-time PCR detection system (Agilent, USA). Each 25 μl reaction mixture contained 12.5 μl of 2 × SYBR Green PCR Mix (TaKaRa, Japan), 1 μl of each primer (20 μM) (Table 1), 1 μl of sample DNA and 9.1 μl of sterile deionized water. The different bacteria did not share the same amplification program. For *Streptococcus pneumoniae*, the procedure was set as one cycle of 95°C for 30 s and then 40 cycles of 95°C for 5 s, 63°C for 30 s, and 72°C for 50 s. For *Klebsiella pneumoniae*, the procedure involved one cycle at 94°C for 5 min, 40 cycles at 94°C for 45 s, 54°C for 60 s, and 72°C for 45 s. For *Pseudomonas aeruginosa*, the procedure involved in one cycle of 95°C for 3 min, 40 cycles at 95°C for 15 s, 55°C for 30 s, and 72°C for 30 s. To obtain the melting curve, an extra cycle was performed: 95°C for 1 min, 55°C for 30 s, and 95°C for 30 s. A tenfold dilution series of plasmid DNA containing target species DNA was used in each real-time PCR assay to generate standard curves for quantitation of target DNA in test samples. The correlation coefficient values of the standard curves were limited from 0.99 to 1.0. Plasmid standards and samples were assayed simultaneously in three parallel PCR reactions.

**Table 1:** **16S rRNA PCR primers used in real**-**time PCR**

Bacterium	Primer	Sequence (5’- 3’)	References
*Streptococcus pneumoniae*	F	ACG CAA CTG ACG AGT GTG AC	[18]
	R	GAT CGC GAC ACC GAA CTA AT	
*Klebsiella pneumoniae*	F	GAG GTC GGT GGT TCA AGT C	[19]
	R	TCG CAG TAA AGA TGG TGG AG	
*Pseudomonas aeruginosa*	F	ATG GAA ATG CTG AAA TTC GGC	[20]

## Results

### DGGE profiles analysis

**Figure 1:**
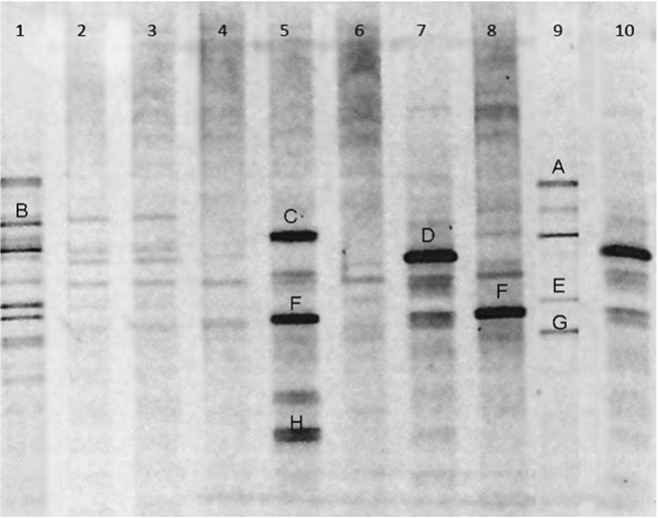
**V3 region of 16S rRNA gene profile from COPD group** **(lane 4–** **10)** **and healthy group** **(lane 1–** **3)** **analyzed by DGGE.** Bands Bands A, B, C, D, E, 5 F,8 F,G and H were cut for sequencing.

**Figure 2:**
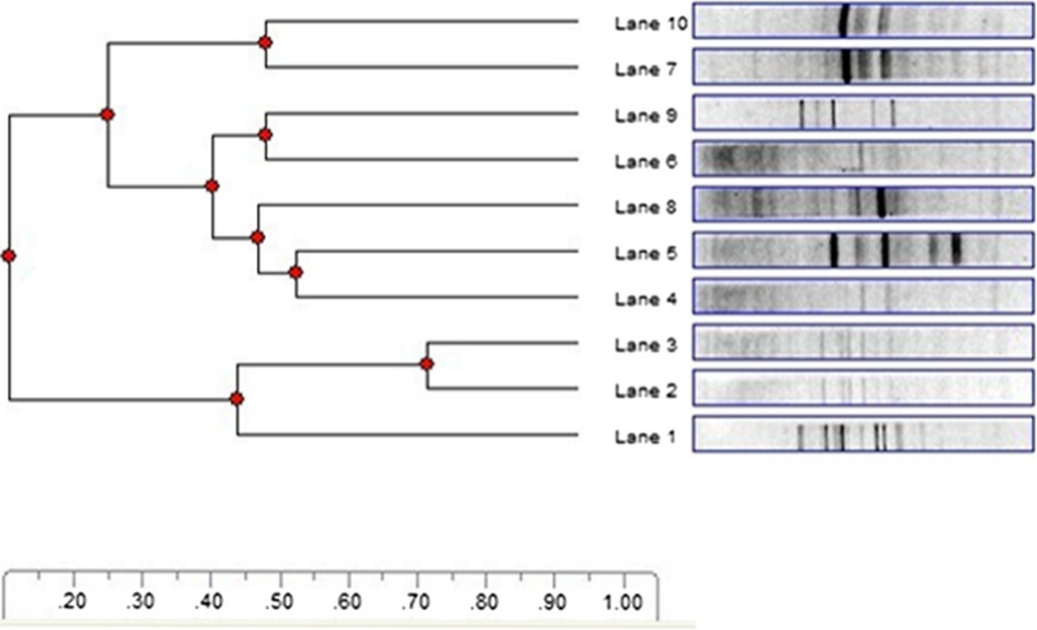
**Dendrogram of DGGE profiles analyzed by UPGMA method** **(COPD group:** **lane 4–** **10;** **healthy group:** **lane 1–** **3).**

The dominant respiratory microbiota of the COPD and healthy group were show in Figure 1. Lanes 4–10 were samples from the COPD group, whereas lane 1–3 represented those from the healthy individuals. The diversity of respiratory microbiota from two groups was analyzed with the Mann–Whitney U test to compare the Shannon–Weaver indexes of diversity (H’) of the bands from DGGE profile. It was clearly demonstrated that the diversity in the COPD groups significantly increased compared with healthy groups (*P* < 0.05). The number of bands was richer in the COPD group with a P value of 0.002 by the Mann–Whitney U test. The dendrogram was constructed based on analysis of similarity score and cluster from DGGE profiles by Phoretix 1D software (Figure 2). Two groups formed significant clustering profiles. There were two main clusters in the dendrogram. One was lane 1–3, related to the healthy group, and the other was lane 4–10 involved in the COPD group. The average number of bands, H’ and evenness (E) between the two groups was listed in Table 2. Overall, the respiratory microbiota communities from the COPD group had their own characteristics that were different from those of the healthy group.

**Table 2:** **Microbiota diversity index analysis of COPD and healthy group**

Diversity index	COPD group (Mean ± SD)	Healthy group (Mean ± SD)	***p*** value
Number of bands (S)	14.43 ± 1.29	7.75 ± 0.25	0.002
Shannon–Weaver index (H’)	2.29 ± 0.24	1.85 ± 0.19	0.013
Evenness (E)	0.84 ± 0.09	0.96 ± 0.02	0.035

### Sequence analysis

Nine bands were selected and cut from the two groups for sequencing in DGGE gels based on quantity analysis (Figure 1). The obtained sequences were further analyzed using a GenBank (NCBI) BLAST search (Table 3). To verify the resolution of DGGE, two adjacent bands from different lanes (5 F, 8 F) were cut and sequenced, and the sequences indicated that both belonged to the *Acinetobacter calcoaceticus*, indicating that the DGGE gel separated V3 16S rRNA genes from different bacteria effectively. As shown in the black frame from DGGE profile, the COPD group shared a band at the same position, whereas there was nearly no band at the corresponding place in the healthy group. Some separated and strong bands (A, B, C, D, E, G, and H) were identified as *Facklamia hominis*, *Prevotella melaninogenica*, *Zymomonas mobilis subsp. Mobilis*, *Nitrosospira multiformis*, *Streptococcus sp*., *Bacteroides vulgatus*, *and Veillonella sp*.

**Table 3:** **Sequence identities of PCR amplicons derived from DGGE gels**

Selected band	Blast result	Bacteria phylum	Similarity (%)
A	*Facklamia hominis*	*Firmicutes*	96%
B	*Prevotella melaninogenica*	*Bacteroidetes*	95%
C	*Zymomonas mobilis subsp. mobilis*	*Proteobacteria*	100%
D	*Nitrosospira multiformis*	*Proteobacteria*	100%
5 F, 8 F	*Acinetobacter calcoaceticus*	*Proteobacteria*	99%
E	*Streptococcus sp*.	*Firmicutes*	99%
G	*Bacteroides vulgatus*	*Bacteroidetes*	100%
H	*Veillonella sp*.	*Firmicutes*	99%

### Real-time PCR analysis

*Streptococcus pneumoniae*, *Klebsiella pneumoniae*, and *Pseudomonas aeruginosa* were chosen as typical important pulmonary bacterial groups for further quantitative ananlysis. *Streptococcus pneumoniae* is a facultatively anaerobic Gram-positive bacterium that normally resides harmlessly within the human nasopharynx but is a major cause of morbidity and mortality worldwide, causing pneumonia. *Klebsiella pneumoniae* is a Gram-negative bacterium. It is facultatively anaerobic. *K. pneumoniae* is an important cause of human infections. Infections or diseases are usually nosocomial or hospital-acquired. The diseases caused by *K. pneumoniae* can result in the death of patients who are immunodeficient. *Pseudomonas aeruginosa* is a Gram-negative bacterium. *Pseudomonas aeruginosa* is an opportunistic human pathogen. It is always listed as one of the top three most frequent Gram-negative pathogens and is linked to the worst disease outcomes. The copy numbers of each bacterium are shown in Table 4. *Streptococcus pneumoniae*, *Klebsiella pneumoniae* and *Pseudomonas aeruginosa* were increased significantly in the COPD group compared with the healthy group (*P* < 0.05). The data were reported as the average estimate of the logarithms of sputum PCR target genetic amplicon copy numbers present in 1 μl of sputum. Results with *P* < 0.05 (Mann–Whitney U test) were considered significantly different.

**Table 4:** **Quantitation analysis of bacterial populations in the COPD and healthy group by real**-**time PCR**(**Mean** ± **SD**)

Bacterium	COPD group	Healthy group	Mann-Whitney T test ***(p)***value)
*Streptococcus pneumoniae*	9.48 ± 3.42	1.24 ± 1.78	0.001
*Klebsiella pneumoniae*	34.65 ± 31.47	6.26 ± 17.69	0.047
*Pseudomonas aeruginosa*	6.19 ± 1.81	1.44 ± 3.22	0.008

## Discussion

According to our results gained from PCR-DGGE profiling techniques, there were multitude pathogens present in all of the samples, which indicated the high sensitivity of this molecular-based approach. Most of the detected microorganisms were catalogued as uncultured bacteria. However, certain most frequently isolated bacteria were not found, such as *Haemophilus spp* and *Moraxella catarrhalis*. In contrast to the routine culture-based approach, in which we usually isolate individual pathogen, it is quite easy to detect multitude microbial pathogens at a high frequency. This notable phenomenon implies that if we intend to define a given respiratory system disease as a polymicrobial infection similar to the cystic fibrosis-associated bronchiectasis, we can employ the more sensitive molecular-based techniques.

With the sputum culture method, most prevalent bacterial isolation was *Pseudomonas aeruginosa*, which was also identified with PCR-DGGE profiling techniques. This bacterium is able produce mortality in some other serious diseases [21]. A prior investigation suggested that the occurrence of the acute exacerbations did not appear to be correlated with the colonization of *Pseudomonas aeruginosa* that much. However, the patients who were colonized with *Pseudomonas aeruginosa* did visit hospitals much more than people who were colonized with *Haemophilus influenza* or some other bacteria [22]. In addition, the detection of *Pseudomonas aeruginosa* in adult samples is much more frequent compared with child samples [23].

Many previous studies have demonstrated that there are some considerable individual variations in the microbial community composition amongst patients. Thus, in our study, we modeled the individual responses to time, with patient as a random effect. As the data had been derived from repeated measures, which was from a subgroup of the sample population. Thus, it provides more accurate information on the underlying polymicrobial infection in sputum samples.

Currently, to analyze bactrial diversity, PCR-DGGE fingerprinting and high-throughput pyrosequencing (NGS) were two important and useful methods, which could matched each other as two molecular analytical methods. Just like all other microbiology methodologies, PCR-DGGE fingerprinting, which is a conventional molecular ecological approach, are not free from drawbacks. It has been reported that DGGE only detected the predominant microbiota and its separation has a bias against low-abundance taxa in the community [24]. Muyzer et al. indicated that only the bacterial species that accounted for more than 1% out of the total microorganism prevalence were identifiable on the DGGE gel [25]. Even so, DGGE was currently considered as one of the few techniques that allowed reproducible visual comparisons of profiles from microbial communities and were successfully applied to a wide variety of microbial ecosystems [26, 27]. Pyrosequencing provided a high-throughput approach to analyze the 16S rRNA gene sequences and explore bacterial diversity in different microhabitats deeply, which can compensate for the disadvantage with the PCR-DGGE method in detecting minor populations in microbiota [28]. This technique has been successfully used in various ecosystems including fermented seafood [29], skin [30], chronic wounds, oral microbiota [24] and so on.

In our study, we just aimed to better estimate the diversity of the sputum community of the healthy and COPD people, and to identify the key population changes relevant to COPD. We first utilized PCR-DGGE with broad range primers that correspond to the bacterial 16S rRNA hypervariable V3 region to investigate the predominant sputum microbiota in these populations. We also quantified the abundance of bacterial subgroups that associated significantly with COPD using quantitative PCR (qPCR). These methods are appropriate to achieve our above purpose. Also, from other related studies, researchers proposed that PCR-DGGE analysis could be used to monitor the dramatic shift of bacterial transition and routinely defined diseases in laboratory [31]. It’s economic and convenient. So in some research area for the large-scale screenning of specific bacteria, this mehtod still occupy the important position and widely used.

Recently, little work has been performed to examine the contribution of the lung or lower respiratory tract microbiome on the pathogenesis of pulmonary diseases. Especially in inflammatory lung diseases such as asthma and COPD, the local microbiome may play an important role in the pathogenesis [32]. Purcell etc. analysed polymicrobial airway bacterial communities in adult bronchiectasis patients in sputum samples by culturing and pyrosequencing approaches [33]. Also there are studies about murine lung microbiome in relation to the intestinal and vaginal bacterial communities by culturing and pyrosequencing approaches [32]. Advancements in next generation sequencing technology have provided means for the comprehensive profiling of the microbial community in the respiratory tract in both physiological and pathological conditions. Alexa describe the COPD lung microbiome of 22 patients with moderate or severe COPD compared to 10 healthy control patients. The composition of the lung microbiomes was determined using 454 pyrosequencing of 16S rDNA found in bronchoalveolar lavage fluid (BALF) [34]. The results showed a significant increase in microbial diversity with the development of COPD. The main phyla in all samples were Actinobacteria, Firmicutes, and Proteobacteria. Although our study hold different experimental purposes, adopted different samples and methods compared with this investigation, the results obtained are the same in some degree.

In a summary, for clinical prevention and diagnosis of COPD in the process of introducing this new respiratory tract flora observed targets. Improve the clinical type of high-maintenance rate of pathogenic bacteria, the medication for instruction, is of great significance to improve the treatment effect of COPD.

## Conclusion

This study have provided a relatively comprehensive picture of our current knowledge of the community structure of the COPD sputum bacterial ecosystem and revealed strong relationship between alterations of sputum microbiota and COPD. By determining the content of several types of potential pathogenic populations, we can provide evidence to aid in the diagnosis and treatment of COPD. It is therefore necessary to further study the variations of the respiratory microbiota with the development of COPD.
